# The nutritional care of people living with dementia at home: A scoping review

**DOI:** 10.1111/hsc.12540

**Published:** 2018-01-24

**Authors:** Louise Mole, Bridie Kent, Rebecca Abbott, Chloë Wood, Mary Hickson

**Affiliations:** ^1^ Institute of Health and Community School of Health Professions Plymouth University Plymouth UK; ^2^ Collaboration for Leadership in Applied Health Research and Care South West Peninsula (PenCLAHRC) The National Institute for Health Research (NIHR) Plymouth UK; ^3^ School of Nursing and Midwifery Plymouth University Plymouth UK; ^4^ Centre for Health and Social Care Innovation Plymouth University Joanna Briggs Institute Plymouth UK; ^5^ Medical School University of Exeter Exeter UK

**Keywords:** community care, dementia, nutritional care

## Abstract

There are an increasing number of people with dementia living in their own home for longer, often supported by a family member. The symptoms of dementia can affect an individual's nutritional status, which can lead to a reduced quality of life for the person with dementia and their family members. A scoping review was conducted from July 2016 until September 2016, using a recognised framework, to explore what is currently known, and identify any gaps in the research regarding the nutritional care of people living with dementia at home. This included any interventions that may have been trialled or implemented, and the views of those living with dementia, carers and clinicians. Six electronic databases were searched from inception to July 2016. A review team was involved in screening and data extraction for selected articles. Published qualitative and quantitative studies were included that explored the nutritional care of people living with dementia at home. Methods included data extraction and conventional content analysis. Stakeholders were involved in the development of final categories. Following screening, 61 studies reported in 63 articles were included. Most studies were cross‐sectional (*n* = 24), cohort (*n* = 15) or qualitative (*n* = 9). Only three were randomised controlled trials. Three overarching categories represented the results: Timely identification of nutritional risk and subsequent regular monitoring of nutritional status, multi‐component tailored interventions and the influence of the care‐giving dyad on nutritional status. Many studies identify people living at home with dementia as a vulnerable group prone to malnutrition; however, a lack of interventions exists to address the increased risk. There is a lack of research exploring the role of home care providers and healthcare professionals in the provision of nutritional care. Further research is required to explore how the emotional aspect of the care‐giving dyad influences nutritional care.

**What is known about this topic**

- People with dementia are at increased risk of malnutrition.
- People living with dementia at home are often reliant on family or professional support to ensure that they maintain adequate nutritional status.
- There are many symptoms of dementia that can increase the risk of malnutrition, if not managed.

**What this paper adds**

- There are a lack of studies exploring how nutritional care for people living with dementia at home could be improved, and the role that care providers have.
- Family carers require support to enable them to identify malnutrition risk, and take action to prevent nutritional decline.
- Healthcare professionals should consider the nutritional care of the family caregiver and the person with dementia (“care‐giving dyad”).

## 1 BACKGROUND

There are an estimated 850,000 people in the UK, and 5.4 million Americans living with dementia (Alzheimer's Association, 2016; Martin, Emiliano, Maelenn, & Matthew, 2014). Symptoms of the different types of dementia (e.g. Alzheimer's disease and vascular dementia) that can affect nutritional status include changes in memory, motor skills, visuospatial ability, taste, appetite and swallow function (Ikeda, Brown, Holland, Fukuhara, & Hodges, 2002; Kai et al., 2015). The presentation of the aforementioned symptoms varies among individuals as the disease progresses (van der Linde, Dening, Matthews, & Brayne, 2014). At least 67% of people living with dementia in the US and the UK live at home, with an estimated 670,000 family and friends providing care in the UK (Martin et al., 2014) and 15 million in the US (Alzheimer's Association, 2015). This role includes meeting the individual's health, emotional and social needs, which become more complex and demanding as the dementia progresses, and can have profound impacts on the individual and their family (Fauth & Gibbons, 2014).

Maintaining an individual's nutritional status including preventing unintentional weight loss (fat and muscle), and meeting fluid and micronutrient requirements, is particularly important in dementia. A decline in nutritional status positively correlates with a decline in cognition and vice versa (Lee, Cheong, et al., 2009; Spaccavento, Del Prete, Craca, & Fiore, 2009). Nutritional decline can begin in the early stages of the disease, which if not addressed can increase the rate of deterioration, as well as increasing clinical vulnerability, e.g. risk of falls, infections and pressure sores (Stewart et al., 2005). Nutritional care in this context, relates to the care provided to people living at home with dementia, in ensuring an adequate intake of energy, protein and other nutrients (Jyvakorpi, Puranen, Pitkala, & Suominen, 2012). The management of symptoms relating to dementia will vary dependent on the care setting. Previous systematic reviews in this area have focused on randomised controlled trials (RCTs) carried out across all care settings (residential care facility, hospital ward environments and own homes), but to date have included minimal analysis of studies specific to “own home” (Abdelhamid et al., 2016; Bunn et al., 2016). Carer surveys have indicated that there is a need for increased primary care support relating to the nutritional needs and consequences associated with dementia in those living at home (Alzheimer's Society, 2012). Community‐based home or domiciliary care (home care providers) may form part of the support that the person with dementia receives, and therefore plays a significant role in helping maintain an adequate nutritional status. Best practice guidelines have been published to support managers of UK domiciliary care agencies (Skills for Care, 2014); however, there is limited literature that evaluates the nutritional care that these agencies provide.

This scoping review sought to answer: what is known about managing the nutritional status of people with dementia living at home? Additionally, the review also explored:

- What interventions have been trialled in this setting to improve or maintain nutritional status?
- What are the difficulties with maintaining and/or preventing decline of nutritional status experienced by people with dementia who live at home?
- What is known about the nutritional consequences of dementia by people living with dementia, family caregivers, home care providers and healthcare professionals?
- Where would carers of people with dementia living at home (family caregivers and home care providers) go to seek help regarding difficulties with eating and drinking?

For the purposes of this scoping review, healthcare professionals refer to any paid worker that interfaces with people with dementia at home, including but not limited to occupational therapists, general practitioners, dietitians and social workers.

## 2 METHODOLOGY

Scoping reviews are useful for gaining a comprehensive overview of the research field of interest. Furthermore, scoping reviews help in mapping the nature and extent of research activities and provide a rigorous and transparent methodology (Levac, Colquhoun, & O'Brien, 2010).

The present study used the Arksey and O'Malley (2005) scoping review framework, which includes identifying the research question, searching for relevant studies, selecting studies, charting the data, and collating, summarising and reporting the results, and consultation with stakeholders to validate findings and facilitate opportunities for knowledge transfer and exchange. This framework is suitable for the inclusion of a range of study types to answer a range of questions related to a broad topic.

A full scoping review protocol was written and agreed by the research team (available from corresponding author on request). Studies included those that featured any intervention, using any study design, with the primary aim of maintaining or improving the nutritional status of individuals (no age restriction, male and females) with dementia or mild cognitive decline, who live at home alone, with family caregivers and/or with home care providers. No restrictions were imposed on the measurement type of outcomes, providing they were focused on nutritional status. Studies focusing on mild cognitive decline were included due to the potential transferability of intervention outcomes with dementia. Studies were also included where a person with dementia, family caregiver, home care provider or healthcare professional's knowledge of nutrition and dementia and awareness of available support was explored, as well as any studies that investigated the nutritional consequences of dementia.

Any studies that were carried out in other healthcare settings (including those focused on homeless populations) were not in the scope of this review. Where studies examined multiple participant groups (e.g. residential care, acute care), only results specific to participants living at home were included and if results were merged across sectors, the study was excluded. Furthermore, any studies concerned with the prevention of dementia were excluded, as well as any studies not written in the English language due to the resource implications of translation (only two were excluded on this basis). The reviewing team agreed to exclude conference abstracts, editorials and opinion pieces as part of the iterative process advocated by Arksey and O'Malley (2005). Authors of included abstracts of interest were contacted if we could not locate a published paper. This was only required for one text which did not subsequently meet eligibility criteria. The scoping review commenced in July 2016.

A search strategy was developed in consultation with an information specialist. The strategy included the following key terms: “dementia” (Alzheimer's, mild cognitive impairment) AND “home” (own house, non‐institutionalised, sheltered accommodation, community, domestic) AND “nutrition” (food, meal, breakfast, lunch, dinner, snack, eat, drink, hydration, feeding, diet, vitamin, supplement, ingestion, cooking, appetite), and was adapted for searching each database. CINAHL, MEDLINE (OvidSP), PsycINFO, EMBASE, The Cochrane Library and TRIP were all searched from date of inception (1937, 1946, 1967, 1974, 1996, 1996 respectively) to July 2016. Forward and backward citation searching was conducted on studies exploring interventions.

Returned article title and abstracts were screened according to the aforementioned inclusion criteria, by two independent researchers (L. M. and C. W.), and any disagreements discussed with a third reviewer (R. A.). The same three researchers similarly conducted full‐text screening.

Data extracted for quantitative studies included sample size, study method, outcome measures and recommendations. Data extracted from qualitative studies also included study method as well as any theoretical framework information.

Data synthesis was conducted using a conventional content analysis approach (Hsieh & Shannon, 2005), in which the descriptive content about the population of interest, setting, study methods, primary and secondary outcome measures, and any recommendations made by the authors were coded. Codes were then grouped into a series of sub‐categories, and grouped again to form a smaller number of overarching categories, which represented the content of included studies. The sub‐categories were presented to the project advisory group, which consisted of three stakeholders; a general practitioner, a community dietitian and a family caregiver. The advisory group was formed at the start of the scoping review through local contacts, and met with the lead researcher on two separate occasions to assist in the development of the review, and formation of overarching categories. Each category was discussed in terms of relevance, and how the stakeholders related their own experiences to it. The main overarching categories were then produced, which describe the existing literature regarding nutritional care of people living at home with dementia.

## 3 RESULTS

**Figure 1:**
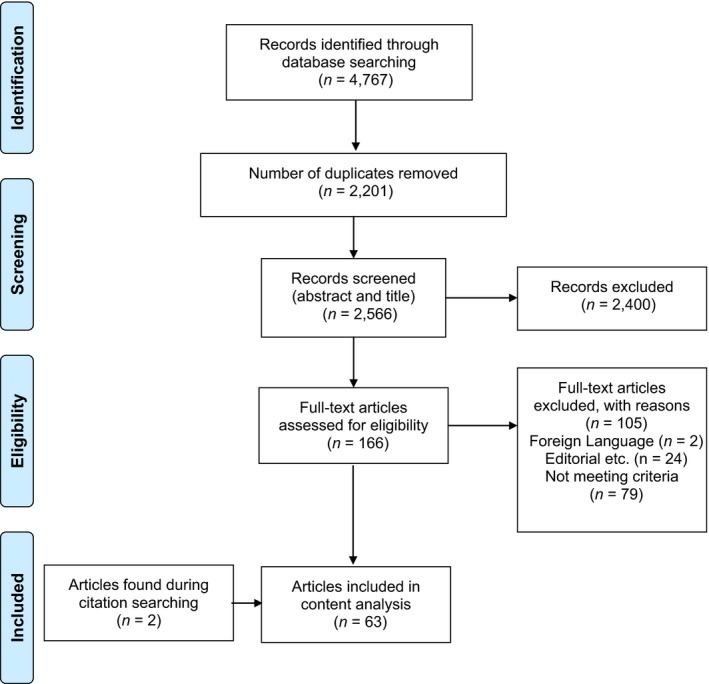
PRISMA diagram showing search results. *N*, number of citations

A total of 2,566 unique articles were retrieved and screened, resulting in 61 studies reported in 63 articles (Figure 1).

Most articles adopted a cohort or cross‐sectional methodology (*n* = 15, *n* = 24), followed by qualitative (*n* = 9), RCTs (*n* = 3), reviews (*n* = 2) and “other” including protocols and pilot trials (*n* = 10) (see Table S1). The studies were most frequently conducted in The Netherlands (*n* = 14), France (*n* = 10), Canada (*n* = 8) and the US (*n* = 6). Included studies focused either on the person with dementia (*n* = 43), the family caregiver (*n* = 6) or both as participants (*n* = 11). Healthcare professionals were the focus of only one study, and no studies involved home care providers. Age of participants ranged from 58 to 84.9 years. Weight loss was the most common primary outcome measure or premise of the study, with a small number concerning micronutrient status. Intervention studies focused on providing nutritional education programmes, tailored nutritional guidance, and the effect of omega‐3 fatty acid supplements on weight and appetite. The majority of included studies restricted the type of dementia diagnosis to Alzheimer's disease, and did not specify the “stage” of dementia, although the Mini Mental State Examination (MMSE) was used as a measure in many studies. The MMSE (Folstein, Folstein, & McHugh, 1975) categorises dementia stages as “mild,” “moderate” and “severe” with overlap between categories. The present scoping review included articles from across the MMSE classification (where stated): mild (*n* = 10), moderate (*n* = 22), a combination of mild to moderate (*n* = 6), moderate to severe (*n* = 1), severe (*n* = 1) and studies that included the whole spectrum (*n* = 4).

The topics and results of included studies generated 26 descriptive codes during data extraction using a content analysis approach previously described (Table 1). These were grouped into eight sub‐categories, which were discussed with the stakeholder group. As a result of the discussion, three overarching interdependent categories were produced. It is worth noting that some codes overlapped between sub‐categories; however for the purposes of this review, the most relevant sub‐category was used. The three overarching categories were: The importance of timely identification of nutritional risk and subsequent regular monitoring of nutritional markers, the need for multi‐component tailored interventions, and the influence of the care‐giving dyad on nutritional status.

**Table 1:** Development of sub‐categories and overarching categories

Initial coding (*n* = 26)^a^	Sub‐categories (*n* = 8)^b^	Overarching categories (*n* = 3)^c^
Supporting carers	The Care‐giving Dyad	The Care‐giving Dyad (Ball et al., 2015; Fjellstrom et al., 2010; Hua‐Chen et al., 2013; Johansson et al., 2011; Keller et al., 2007, 2008; Puranen et al., 2014; Rullier et al., 2013; Shatenstein et al., 2016; Wlodarek & Glabska, 2013)
Caregiver nutritional status is linked with nutritional status of person they are caring for		
Caregiver burden is a risk factor for weight loss		
Caregivers may develop coping strategies		
Caregiver gender		
Increased dependency on the caregiver		
Regular monitoring of nutritional status	Timely identification of malnutrition risk and regular monitoring	Identification and measurement of nutritional status/risk (Annweiler et al., 2012; Bourdel‐Marchasson et al., 2001; Buell et al., 2010; Burns et al., 1989; Chi et al., 2015; De Bruin et al., 2010; De Rouvray et al., 2014; Droogsma et al., 2013; Faxen Irving et al., 2009; Ferrario et al., 1996; Guerin et al., 2009; Guyonnet et al., 1998; Hagnelius, Wahlund, Schneede, & Nilsson, 2012; Ikeda et al., 2002; Keene & Hope, 1998; Kwan et al., 2005; Lyngroth et al., 2015; Milward et al., 1999; Presse et al., 2008; Puranen, Pitkala, & Suominen, 2015; Rivière et al., 1998; Rivière et al., 2002; Salva et al., 2011; Scarmeas et al., 2009; Shatenstein, Kergoat, & Nadon, 2001; Shatenstein et al., 2007, 2008; Silva et al., 2013; Soto et al., 2012; Suominen et al., 2015; Tully et al., 2003; Vellas et al., 2005; Venci et al., 2015; Winograd et al., 1991)
Nutritional status is a risk factor for institutional placement		
Holistic nutritional assessments	Method of measuring/identifying nutritional status	
Measurement of nutritional status		
Micronutrient supplementation	Micronutrient status	
Omega‐3 may play a role for reduced conversion of MCI to AD		
Micronutrient deficiencies		
Hyperphagia	Dietary Intake	
Imbalanced dietary intake		
Suboptimal diet is evident early in the onset of AD		
Beverage contribution towards total energy intake	Energy Balance	
Energy requirements and body composition		
Two types of weight loss in dementia: progressive and severe		
Increased risk of malnutrition	Predictors of malnutrition risk	Tailored nutritional guidance (Bilotta et al., 2010; Buffa et al., 2010; De Bruin et al., 2010; Droogsma et al., 2014; Gillette‐Guyonnet et al., 2000; Guerin et al., 2005; Isaia et al., 2011; Jyvakorpi et al., 2012; Lee, Hong, Cheong, & Oh, 2009; Miyamoto, Higashino, Mochizuki, Goda, & Koyama, 2011; Nes et al., 1988; O'Neill, McKiernan, Gibney, Walsh, & Coakley, 1990; Riviere et al., 2001; Salva et al., 2009; Smith et al., 1998; Tombini et al., 2016; Wolf‐Klein et al., 1995)
Degree of physical impairment is a risk factor for malnutrition		
Cognitive impairment may predict malnutrition risk		
Nutritional education programmes	Multi‐component, tailored nutritional guidance	
Tailored nutritional guidance		
Multi‐component interventions		
Day care facilities that involve attendees in food preparation can increase dietary intake		

a Twenty‐six initial codes were noted while reviewing included studies.

b These were then grouped into eight sub‐categories, and following discussions with stakeholders.

c Three overarching categories.

### 3.1 Category 1: Identification of nutritional risk and regular monitoring of nutritional status

Studies aligned with this category (*n* = 35) included those focussed on methods of identifying and measuring nutritional status or risk (including micronutrient status), measurement of dietary intake and energy balance, and the timely identification and ongoing monitoring of nutritional risk.

There is no consensus as to the most appropriate screening or assessment tool for people with dementia. Most studies used the Mini Nutritional Assessment (MNA) or Short Form Mini Nutritional Assessment (SF‐MNA) (*n* = 16), which seemed appropriate for participants with dementia, as they contain specific questions regarding neurophysiological problems, as well as capturing high‐level information regarding quantity of food and fluids consumed, and mode of feeding (Vellas et al., 2006). The Nutrition Screening Initiative (NSI) checklist and the nutritional form for elderly people were used in two studies. No included study used the Malnutrition Universal Screening Tool (MUST) (BAPEN, 2016). It is notable that no malnutrition screening tool specific to people with dementia, such as the Edinburgh Feeding Evaluation in Dementia Scale (Watson, Green, & Legg, 2001) was used.

A tendency towards undernutrition and dehydration was identified in participants in the early phases of Alzheimer's disease, which increased as the disease progressed. In this study (Buffa, Mereu, Putzu, Floris, & Marini, 2010), bioelectrical impedance vector analysis was used to measure body cell mass, and the authors propose it as a tool for screening and monitoring nutrition and hydration status in Alzheimer's disease. In contrast, two other studies using anthropometric and biochemical measures found no significant differences in the nutritional status of participants with dementia. One study investigated a group with dementia compared to a control group without dementia representing the general older population over 1 year, and the other study focussed on a group of people with dementia who were assessed at baseline and after 6 months (Burns, Marsh, & Bender, 1989; Ferrario et al., 1996).

Alternative predictors of reduced nutritional status included the clock‐drawing test (Lyngroth, Sorensen, Madsen, Soderhamn, & Grov, 2015) and serum vitamin C levels (Rivière, Birlouez‐Aragon, Nourhashémi, & Vellas, 1998). One study associated inflammatory markers with body weight change, finding it inversely correlated with plasma tumour necrosis factor (TNF) (Kwan, Kwok, Lam, Woo, & Chiu, 2005). People living at home with dementia were found to have reduced serum markers of vitamin D (Annweiler et al., 2012; Buell et al., 2010), plasma vitamin E and retinol (Bourdel‐Marchasson et al., 2001), and serum haemoglobin levels (Milward et al., 1999) compared to control groups. Increased incidence of behavioural and psychological symptoms of dementia was proposed as precursors to rapid weight loss (Guerin et al., 2009).

Dietary intake of a variety of macro and micro‐nutrients was the focus of some studies (*n* = 8) included in this review. The overall dietary intake of participants with Alzheimer's disease in two separate studies was poor compared to cognitively intact age‐matched controls, and the authors suggest that this led to a decline in body weight (Guyonnet et al., 1998; Shatenstein, Kergoat, & Reid, 2007). Both studies also noted a lack of routine nutritional assessments being conducted at outpatient clinics, a factor re‐iterated by many studies included in this scoping review. Hyperphagia (“over‐eating”) was the primary subject of investigation in three studies of people with dementia (Chi, Lin, Chang, & Wu, 2015; Keene & Hope, 1998; Smith, Vigen, Evans, Fleming, & Bohac, 1998). Hyperphagia was associated with increased functional decline; however, authors suggested that hyperphagia may be beneficial by promoting energy balance in people whose energy expenditures are increased (e.g. due to agitation). One study focused on beverage consumption, which was found to contribute towards micronutrient intake, but provided minimal contribution towards average energy intake (~12.5%) in older adults with memory decline (Venci, Hodac, Lee, Shidler, & Krikorian, 2015).

The average daily dietary intake of Vitamin K was explored in one study, and found to be less in a community‐dwelling group of participants with early‐stage Alzheimer's disease compared to a control group, which was attributed to a reduced dietary intake of green vegetables by the Alzheimer's group (Presse, Shatenstein, Kergoat, & Ferland, 2008). The same study also measured overall daily energy intake between groups, and found that the Alzheimer's group consumed ~293 kcals less than the control group. One study found that the consumption of a Mediterranean diet, which is rich in green vegetables, was associated with a reduced risk (48%) of progression from mild cognitive impairment to Alzheimer's disease (Scarmeas et al., 2009); however, the biological mechanisms producing this protective effect remain unclear.

Studies that measured the nutritional status of participants recommended that undernutrition risk should be identified as early as possible—using an appropriate tool, but none specified the best tool to use. The studies also suggested that nutritional status should be monitored regularly thereafter (Guerin et al., 2005; Isaia et al., 2011; Tombini et al., 2016); no firm data are available for frequency but one paper recommends every 6 months (Guerin et al., 2009). Most studies did not specify who should be responsible (i.e. family carer or healthcare professional) for identifying or monitoring the nutritional status of people living with dementia at home. Healthcare professionals are, however, best placed to identify or monitor the nutritional status of both family carer and person with dementia (Tombini et al., 2016).

### 3.2 Category 2: Multi‐component tailored interventions

Providing nutritional education to caregivers of people living at home with dementia was trialled in three RCTs (Riviere et al., 2001; Salva et al., 2011; Suominen et al., 2015) all of 1‐year duration, and a quasi‐experimental study of 6‐month duration (Shatenstein, Kergoat, & Reid, 2016). The interventions were all “multi‐component,” and consisted of two or more of the following: carer group education sessions, physician training, carer advice leaflets, dietitian home visits with individual nutritional plans, micronutrient supplementation and oral nutritional supplementation. Two of the RCT studies reported a statistically significant increase in nutritional status (measured by MNA) in the intervention groups compared to the control groups (Riviere et al., 2001; Salva et al., 2011). Protein and calcium intakes increased (at statistically significant levels) in the intervention group of the third RCT study (compared to the control group); however, there was no difference in weight change (Suominen et al., 2015). The quasi‐experimental study, which measured the effectiveness of the application of clinical dietetic principles as well as increased weight monitoring, did not find an increase in dietary intake of energy, protein or fat in the intervention group (Shatenstein et al., 2016). Multi‐component interventions appear to deliver some beneficial nutritional outcomes, and show potential for supporting the nutritional care of people living at home with dementia. Further intervention trials in this setting are needed to confirm this.

The impact of nutritional status on people living with dementia's quality of life (QoL) was explored in one study, which recruited people with dementia and family carers as participants (Suominen et al., 2015). QoL increased in the intervention group (statistically significant), who received tailored nutritional counselling. In particular, the dimensions that increased included: mental function, breathing, usual activities and depression (measured using the health‐related QoL tool; Sintonen, 2001). As protein intake also increased in the intervention group, the authors suggest that this influenced the increase in QoL, as well as a reduction in the number of falls.

Limitations existed in all intervention studies, which were likely to affect the outcomes. There were inconsistencies with caregiver characteristics between intervention and control groups in three of the four studies, including differences in age, and number residing with the person with dementia. One study included only spousal caregivers, and one did not specify the type of caregiver (only that it included spouse or children of person with dementia). The demographic characteristics of the caregiver are important to understand, as these may influence a number of factors including caregiver burden, knowledge of dementia and symptoms, and awareness of support services (Brodaty & Donkin, 2009). Furthermore, family values, formation and intergenerational relations may vary between age groups (Hoff, 2015). In the studies that documented the dropout rate of participants, this varied between 14% and 34.6%. This may have been due to research participant burden because of the study length and obligations of participants.

### 3.3 Category 3: The care‐giving dyad and the influence on nutritional status

This category was represented in 10 studies, and refers to the effects of physical and psychosocial interactions between a family caregiver and person with dementia and the role this interdependent dyad has on the nutritional status of both parties. No study included in this review focused on the dyadic relationship between healthcare professional or home care provider and person with dementia. Furthermore, the triadic relationship between family caregiver, healthcare professional or home care provider and person with dementia was not explored. Some qualitative studies focused exclusively on the family caregiver and their perceptions of the everyday life aspects of cooking and coping with problematic eating behaviours (Ball et al., 2015; Fjellstrom et al., 2010; Hua‐Chen, Hui‐Chen, & Jing‐Jy, 2013; Keller, Edward, & Cook, 2007). These studies show that the family carers involved felt unsupported and uninformed with respect to the nutrition‐related care of the individuals with dementia, and wanted more input from professionals.

The importance of the caregiver and person with dementia having a trustful relationship was highlighted in one study (Johansson, Christensson, & Sidenvall, 2011), which aimed to capture the self‐description of managing mealtime tasks by people with mild to moderate dementia. The participants of this study demonstrated that maintaining independence was important to them, and they might not express difficulties at mealtimes for fear of losing this. This was the only qualitative study involving people with dementia as the main participants. A gender difference in the ability of spouses being able to cope with adequate nutritional intake for both themselves and their spouses with Alzheimer's disease was observed by one study (Puranen et al., 2014), where male caregivers were reported to struggle more so than female caregivers. Increased caregiver burden was identified as a predictor of weight loss in people with Alzheimer's disease (Bilotta, Bergamaschini, Arienti, Spreafico, & Vergani, 2010; Gillette‐Guyonnet et al., 2000). The nutritional screening result (using MNA) of participants with Alzheimer's was positively associated with the screening result of the caregiver in two separate studies (Rullier, Lagarde, Bouisson, Bergua, & Barberger‐Gateau, 2013; Tombini et al., 2016).

One qualitative study involved healthcare professionals and dementia charity workers as participants, exploring the range of nutritional concerns they had experienced in their work with clients with dementia (Keller et al., 2008). The main concerns included inadequate and imbalanced food intake of clients, inadequate access to food, and maintaining independence with meal preparation and eating.

## 4 DISCUSSION

The purpose of this scoping review was to examine the available research on what is known about the nutritional care of people living with dementia at home, with the further aim of identifying any interventions that have been trialled in this setting. Additional aims included identifying any difficulties associated with maintaining and/or preventing decline in nutritional status experienced by people with dementia who live at home, and the knowledge of nutritional issues associated with dementia by people with dementia, carers and clinicians, including where they would go to seek help. People living at home with dementia at all stages of the disease have been shown to be at increased risk of undernutrition by many studies included in this review. The benefits of identifying nutritional risk as early as possible, and ongoing monitoring of nutritional status have also been demonstrated by many studies. The family caregiver role was examined in the context of the provision of nutritional care to people living with dementia, and the nutritional status of the caregiver and the person with dementia. Poor nutritional status of the caregiver was associated with poor nutritional status of the person with dementia, which may be due to increased caregiver burden, coping strategies and adjustment to the physical and emotional changes associated with dementia.

### 4.1 Interventions trialled in this setting

The three RCTs included in this review focused on the provision of nutritional education to family caregivers and tailored nutritional guidance to people living at home with dementia. The interventions were all “multi‐component,” in that they targeted a range of variables of nutritional care provision (e.g. family carer education, enhanced access to dietetic support and regular weight checks). The interventions delivered small‐scale but positive outcomes. These findings need to be developed to establish and test acceptable and effective ways to support nutritional care. Given the range of symptoms, the variable rate of progression and the complex nature of adequate nutritional intake, it seems likely that successful interventions for this group will be complex and have built in flexibility to adapt to individuals’ needs. Others have concluded that multi‐component interventions appear to have advantages over single‐component interventions including: reducing symptoms of depression, improving QoL, reducing carer burden, and reducing the behavioural and psychological symptoms of dementia (Laver, Milte, Dyer, & Crotty, 2016). Therefore, interventions will need to be developed so that they target multiple determinants, across multiple levels of influence.

### 4.2 Identifying any difficulties associated with maintaining and/or preventing decline in nutritional status

The content analysis presented shows that the key issues relating to nutritional status in people living at home with dementia are identifying nutritional risk accurately, monitoring change regularly, considering not only energy balance but also micronutrient status and the quality of the diet. The most suitable tool for screening for the undernutrition of people with dementia has not yet been explored by any study; however, the SF‐MNA has been shown to more accurately assess malnutrition risk compared to MUST in frail, older hospitalised patients (Slee, Birch, & Stokoe, 2015). Therefore, it may be that a tool specifically tailored to people with dementia is required. Certainly, more work is needed to understand which screening tool will identify those at risk to enable more accurate targeting of interventions. Undernutrition screening tools provide a structured approach across care settings, but should not replace routine observation by carers and healthcare professionals. However, this relies upon these people having knowledge of the nutritional consequences associated with dementia, and this scoping review found little data to support this. In fact, we found no articles investigating what people with dementia or their carers understood about the possible dietary changes that might come with dementia. This is crucial to allow effective monitoring to ensure interventions are working or early identification of deterioration.

Evidence suggests that people with dementia may experience a wide variety of difficulties associated with eating and drinking, ranging from taste changes to reduced appetite, that make them susceptible to unbalanced dietary intake (both macro‐ and micro‐nutrients), resulting in a greater risk of undernutrition (Droogsma, van Asselt, & De Deyn, 2015; Ikeda et al., 2002; Kai et al., 2015). However, family caregivers may lack the information and resources required to assist with these difficulties (Shatenstein, Kergoat, Reid, & Chicoine, 2008), leading to a deterioration in nutritional status. As highlighted previously, caregivers may also lack the knowledge to successfully identify and monitor such changes. An improved understanding of the family caregiver's experience is essential for the development of future nutrition interventions adapted to the needs of older adults with dementia and their caregivers (Silva, Kergoat, & Shatenstein, 2013).

### 4.3 Nutrition knowledge and information sources

In previous sections, we have shown that the knowledge of the family carers appears crucial. Qualitative studies suggest that caregivers recognise the importance of adequate nutrition but feel unsupported and uninformed regarding making changes. Despite this, no studies identified where carers of people with dementia would go to seek help regarding difficulties with eating and drinking. The lack of evidence in this area may be due to the recent expansion of support services available to family caregivers and people living with dementia at home, which have yet to be methodically explored. For example, in the UK setting alone, there are many online forums, social networking channels, charity support workers (local and national), volunteer agencies, health and social care professionals as well as friends and other family members who could provide advice to a caregiver, but no information exists on the content or quality of these sources. Caregivers may choose to seek advice regarding nutritional care from a non‐professional source, due to potential delays in speaking with a healthcare professional, and perhaps due to existing trustful relationships with non‐professionals. However, the information provided may be of questionable quality. No study has assessed the quality of nutritional guidance provided to people with dementia and their carers, whether online or from “informal” networks. Only three qualitative studies included the person with dementia as a participant. This is perhaps due to a focus on the family caregiver role, as they are the people required to make more of the decisions regarding nutritional care as the dementia progresses. Nevertheless, there exists a gap in the literature relating to the knowledge, experiences and views held by the people living with dementia themselves.

The role of home care providers was not explored in any study. In the UK, the quality of care provided to people with dementia living in their own homes has only recently become a focus, following a survey conducted by the Alzheimer's Society (Carter, 2016). This highlighted the major issues regarding home care as home care providers lacking knowledge of the condition and how to adapt care provision, and only 2% of people living with dementia feeling that home care workers have enough dementia training (Carter, 2016). In the US, a significant gap has been highlighted in the demand for healthcare workers who are trained to care for older adults and those choosing this as a career (Alzheimer's Association, 2015). A recent review has highlighted the potential benefits of including family caregivers and home care providers as part of malnutrition screening, and having a role in the delivery of nutritional interventions for older adults (without dementia) living in the community (Marshall, Agarwal, Young, & Isenring, 2017). These findings may also translate to people with dementia, to improve nutritional status of care recipients and improve the QoL for the caregiver.

Healthcare professionals also have an important role in the provision of nutritional care to people living with dementia at home. There is, however, a lack of evidence exploring how different healthcare professionals perceive the importance of nutritional care in this group, and their role in the identification of undernutrition risk and subsequent nutritional care. Diagnostic guidelines are constantly evolving, and undernutrition screening is not routinely carried out in standard practice. There are many additional health factors to consider when supporting someone with dementia, therefore nutritional care may not be an immediate priority. Some healthcare professionals may also take a “nihilistic” view as nutrition will not influence the prognosis (Iliffe et al., 2009).

There is limited evidence regarding the impact of overall nutritional status on QoL for people living at home with dementia. A shortcoming of current QoL measurement tools is that they are not sensitive to changes over time, which is critical to evaluating participant response to treatment and determining the effects of dementia progression on QoL (Ready & Ott, 2003). Therefore, the relationship between nutritional status and QoL of people living at home with dementia warrants further investigation.

There is a lack of studies that have explored the emotional significance of the dyad relationship in the context of nutritional care provision, with most interventions focusing on manipulating the physical elements (e.g. improving oral intake, reducing weight loss and caregiver education). This suggests that any intervention may need to be “dyadic,” in that it involves both parties. Previous reviews suggest that this approach has produced positive outcomes in future care‐planning interventions in terms of relationship quality and social relations, as well as providing essential information to healthcare professionals when developing such plans (Braun et al., 2009; Moon & Adams, 2013). The social aspect of mealtimes should also be considered in the context of the care‐giving dyad. A “family‐style” dining environment in care homes (including eating with caregivers and familiar music being played during mealtimes) has been found to increase oral intake, body weight and QoL (Bunn et al., 2016). Keeping routines (such as mealtimes) familiar for a person with dementia may evoke feelings of social “security” and therefore maintain adequate oral intake (Brittain, Corner, Robinson, & Bond, 2010). Future interventions aimed at improving nutritional care for people living at home with dementia, should take into account the influence of such human factors alongside physical factors such as meal composition (Keller et al., 2007).

### 4.4 Limitations

There are a few limitations worth noting. As studies that involved mixed populations (dementia and non‐dementia) and mixed settings were excluded, some relevant information may have been missed. Inconsistent terminology used in different countries for assisted home living, may have resulted in some relevant studies being excluded. Two non‐English language papers were excluded; however, these are unlikely to substantially alter the overall conclusions. The intention of this scoping review was to provide a comprehensive overview of the research pertaining to a broad topic. The assessment of quality of included studies was not conducted, so recommendations have not been made for specific interventions that may promote improved nutritional status in people living at home with dementia.

## 5 CONCLUSION

Supporting the nutritional needs of people living with dementia in their own homes should be an important component of holistic primary care provision. The nutritional consequences of dementia can have profound impacts upon the individual, family caregiver's and primary healthcare services. Although many studies have highlighted the increased risk of undernutrition in this group, very few interventions have been trialled in this setting to address the issue. There is also a gap in the literature regarding the role that home care providers and healthcare professionals have in providing nutritional care to people living with dementia at home. Families have expressed a need for more information and support regarding nutritional care, and healthcare professionals need to consider how this can be provided in a cost‐neutral manner. The provision of adequate nutrition is reliant upon not just knowledge and ability, but also the emotional influence of the care‐giving dyad. Future intervention studies may need to consider using dyadic approaches to nutritional care provision in the home, which is a novel concept compared to the current patient‐centred approaches to dementia care.

## CONFLICTS OF INTEREST

The authors declare no conflicts of interest.

## AUTHOR CONTRIBUTIONS

L. M., M. H., B. K. and R. A. conceived and designed the study. L. M. completed the searching and with C. W. carried out title, abstract and full‐text screening. R. A. acted as third reviewer. L. M., M. H., R. B., B. K. and C. W. were involved in the data analysis and interpretation, drafting the article and critical revision of the article. All authors gave final approval of the version to be published.

## ETHICAL APPROVAL

Ethics approval was not required for this study.
