# Supplementary material for: The nutritional care of people living with dementia at home: A scoping review
Source: Health Soc Care Community. 2018 Jan 24;26(4):e485–96. doi: 10.1111/hsc.12540 (PMC6849562; doi:10.1111/hsc.12540)
Supplement: Supplementary file 1 [file HSC-26-e485-s001.docx]

| **First Author and (Year)** | **Geographical Area** | **Sample Size and Methodology** | **Aims** | **Time frame** | **Recommendations for future research or clinical practice** |
| --- | --- | --- | --- | --- | --- |
| Ahmed et al., (2014) | Australia | n = 75 Prospective case controlled | Measuring eating habits and hunger/satiety in two frontotemporal syndromes compared with AD and healthy controls. | 24 hours | Relationship between disinhibition, alterations in eating and orbitofrontal cortex integrity. Metabolic profile in FTD vs. amyotrophic lateral sclerosis. Actinography to understand caloric intake vs. expenditure. |
| Andrieu et al., (2001) | France | n = 318 Prospective cohort | Relationship between nutritional status and risk of institutionalisation. | 12 months |  |
| Annweiler et al., (2012) | France | n = 125 Prospective cohort | Low serum 25-hydroxyvitamin D (25OHD) concentrations and association with MCI | 9 months | Further prospective analyses to include more memory clinics and whether low serum Vitamin D could be prognostic amongst of dementia amongst those with mild cognitive impairment. |
| Ball et al., (2015) | Australia | n = 14 Descriptive exploratory study semi-structured interviews | To explore the perceptions of family carers of people with dementia, with emphasis on feeding-related challenges, burden of care, and practical strategies. |  | A need for strategies and services focusing on family carer education. |
| Bilotta et al., (2010) | Italy | n = 105 Prospective cohort study | Whether caregiver burden can be an independent predictive factor of weight loss at 3 months in those with AD living at home. | 3 months | Further longitudinal studies with larger sample sizes and more complete psychological assessments of caregivers. |
| Bourdel-Marchasson et al., (2001) | France | n = 43 Case control study | Investigating blood markers of oxidative stress and antioxidants in normally nourished elderly people with AD. | 1 month | The impact of Vitamin E supplements on weight maintenance and co-morbidity. |
| Buell et al., (2010) | Massachusetts | n = 318 Cross sectional prospective study | To explore mechanisms through which Vitamin D may be involved in neurocognitive function. | 4 years |  |
| Buffa et al., (2010) | Italy | n = 560 Cross sectional study | Bioelectrical Impedance Vector Analysis (BIVA) and its use for screening/monitoring nutrition/hydration in AD. |  |  |
| Burns et al., (1989) | London | n = 78 Case control study | Investigating the nutritional status of people with dementia in hospital, living at home vs. control. | 6 months |  |
| Chi et al., (2015) | Taiwan | n = 104 Cross sectional and correlational | To investigate the prevalence and patterns of and factors associated with hyperphagic behaviour in Taiwanese patients with dementia living at home. | 5 months | Health professionals in home care or in clinics should be educated regarding the prevalence, patterns, and correlates of hyperphagic behaviour. |
| De Bruin et al., (2010) | Netherlands | n = 53 Comparative cross-sectional study | To compare dietary intakes of older people with dementia receiving day care at regular day care facilities (RDCFs) or at green care farms (GCFs). | 19 months | Larger sample sizes, more observations. |
| De Rouvray et al., (2014) | Central Africa | n = 1016 Cross-sectional study | To determine the nutritional status of elderly African people and to investigate the association between undernutrition and dementia. |  | Larger sample size. |
| Droogsma et al., (2013) | Netherlands | n =312 Retrospective cross-sectional study. | To determine the prevalence of malnutrition and its relation to various factors in community-dwelling elderly with newly diagnosed AD. | 10 years | The relationship between daily functioning, nutritional status and AD warrants further investigation. |
| Droogsma et al., (2014) | Netherlands | n/a Systematic Review | Review of the effect of interventions in community dwelling AD pts with risk of undernutrition. |  | Future research should focus on establishing risk factors to design targeted interventions. |
| Droogsma et al., (2015) | Netherlands/Belgium | n/a Review | To summarise existing knowledge about weight loss and undernutrition in com- munity-dwelling AD patients. |  |  |
| Faxen-Irving et al., (2009) | Sweden | n = 204 Randomised Controlled Trial | The effects of omega-3 fatty acid (FA) supplements on weight and appetite in patients with mild to moderate Alzheimer’s disease (AD) in relation to inflammatory biomarkers and apolipoprotein E. | 12 months | Studies exploring the effects of omega-3 on the gene transcription of albumin. |
| Ferrario et al., (1996) | Italy | n = 44  Prospective cohort | Nutritional patterns in the elderly with AD and mild cognitive impairment. | 12 months |  |
| Fjellstrom et al., (2010) | Sweden | n = 17 Focus group interviews | To examine how people living with people with AD perceive everyday life aspects of food choice, cooking, food-related work and nutritional concerns. |  | Both qualitative and quantitative methods are needed with special focus on the themes highlighted in this study. |
| Gillete-Guyonnet et al., (2000) | France | n = 76 Longitutunal cohort | Descriptors of weight loss in AD. | 30 months |  |
| Guerin et al., (2005) | France | n = 395 Prospective study single-centre cohort | To describe progressive and severe weight loss in the course of AD. | 1 year | F/up of people with AD should be every 6 months and include assessments of functional, nutritional, and neuro-psychologic status. |
| Guerin et al., (2009) | France | n = 395 prospective single-centre cohort | To characterise massive weight loss in AD (≥5 kg over 6 months) over a number of years. | mean f/up 2.5yrs (full time 6.5yrs) | Links with BPSD need to be explored further, using large cohorts. |
| Guyonnet et al., (1998) | France | n = 76 Prospective study | Monitoring the incidence and severity of weight loss over time in people with AD living at home. | 1 year | Regular monitoring of nutritional status in AD pts as soon as diagnosis is suspected and weight loss prevention programmes to reduce caregiver burden. |
| Hagnelius et al., (2012) | Sweden | n = 926  Cross-sectional study | Total plasma homocysteine differences in relation to prescribed Vitamin B12 in people with dementia. |  | Regular monitoring of the nutritional status in elderly geriatric patients is necessary. Healthcare workers need to pay more attention to nutrition and compliance. Use of biomarkers early to identify people at risk. |
| Hua-Chen et al., (2013) | Taiwan | n = 13 Semi-structured interviews | Family caregivers experience with problematic eating behaviours amongst community-dwelling older adults with dementia. |  | Hospital based dementia outpatient clinics to engage in disease progression on a regular basis. Care skills of caregivers need to be developed to take these specific behaviours in mind, as well as providing greater psychological assistance. |
| Ikeda et al., (2002) | UK | n = 91  Questionnaires | Investigating the frequency of changes in eating behaviours and development of eating behaviours in frontotemporal dementia and AD. |  | The efficacy of SSRIs in other primary eating disorders also argues for the need for larger placebo controlled trials in frontotemporal dementia. |
| Isaia et al., (2011) | Italy | n = 130  Cross-sectional cohort study | Evaluation of the nutritional characteristics of non-institutionalised people with dementia. |  | Appropriated evaluation of nutritional status could prevent and treat nutrition related problems in people with dementia living at home. |
| Johansson et al., (2011) | Sweden | n = 15 Ethnography | Capturing the self-description of managing mealtime tasks by persons with dementia. |  | It is important for caregivers to create a trustful relationship even before problems arise to be able to support the persons when necessary. |
| Jyvakorpi et al., (2012) | Finland | n = 202  Randomised controlled trial | Whether an individualised nutritional care intervention has an effect on weight, health, physical functioning, and Quality of Life in those with AD and their spouses living at home. | 1 year |  |
| Keene & Hope., (1998) | UK | n = 104 Prospective longitudinal study | Investigation of hyperphagia and its elements during the course of dementia. | 1 year (some until death) |  |
| Keller et al., (2007) | Canada | n = 23 Descriptive qualitative semi-structured interviews | Family caregiver’s perspectives on mealtimes when caring for someone with dementia. |  | Some formal education can help change attitudes about the primary goals of mealtimes (eg, connection and pleasure), but innovation, experimentation, and flexibility are needed to identify how best to meet the eating challenges they encounter at mealtimes. |
| Keller et al., (2008) | Canada | Study 1: n = 14 interviews with formal providers Study 2: n = 74 email survey | Nutrition education needs and resources for dementia care in the community. |  | Front-line staff require more nutrition education than currently provided. |
| Kwan et al., (2005) | Hong Kong | n = 81 Pilot prospective study | To identify associated factors for weight changes in AD pts. | 6 months | The weight loss associated with AD can be prevented and ameliorated by dietary manipulation and caregiver training. Drugs that can lower plasma TNF concentrations may be beneficial to those subjects with AD who fail to gain weight despite dietary intervention. |
| Lee et al., (2009) | South Korea | n = 490 Cross sectional study | Comparing differences in nutritional risk between mild cognitive impairment groups and normal cognitive function elderly groups in the community. |  | Screening for nutritional risk should be included in geriatric evaluations. |
| Lyngroth et al., (2015) | Norway | n = 213 Cross sectional study | Association between people with dementia and caregivers self-reports of nutritional status. |  | Self-reporting and proxy-rating seem both applicable for nutritional screening among moderate cognitive impaired. Reduced MMSE and/or failed clock drawing test might predict the risk of undernutrition. |
| Milward et al., (1999) | Australia | n = 316 Retrospective cross sectional study | The association of coincident anaemia with different dementias in elderly, community-dwelling people. |  | Larger community-based investigations of the associations between anaemia and dementia are needed. |
| Miyamoto et al., (2011) | Japan | n = 60 Cross sectional observation study | Examination of whether dementia-related eating difficulties increase probability of weight loss in the elderly with dementia who attend day-centres. | 6 months | The relevance of the Eating Behaviour Scale (EBS) and its association with weight loss in this group need to be further assessed in a larger population. Standardised methods of assessing eating behaviours in subjects with dementia should be developed. |
| Nes et al., (1988) | Norway | n = 16 Cross sectional study | Examination of dietary intakes and blood levels of certain nutrients in a non-institutionalised group with dementia vs. healthy control group. |  | 10 micrograms Vitamin D may be necessary to ensure satisfactory 25-OH-Vitamin D level for persons above 75 yrs. |
| O'Neill et al., (1990) | Ireland | n = 18  Cross sectional study | Dietary and anthropometric measures in mild to moderate AD in community-dwelling people. |  | Further studies are needed to determine whether low bodyweight is associated with the aetiology of the disease or whether it is a result of the disease process. |
| Presse et al., (2008) | Canada | n = 62 Cross sectional study | Dietary Vitamin K intake in community-dwelling people with early-stage AD vs. control group |  | Future longitudinal studies need to consider Vitamin K and AD, and the role of diet in AD. Dietitians should be mindful of the role of green vegetables in cognitive health. |
| Puranen et al., (2014) | Finland | n = 99 (caregiving dyads) Cross sectional study | Clarification of the association of the caregiver’s sex on the nutrient intake of AD couples. |  | A need exists for tailored nutritional guidance amongst older individuals and especially among male caregivers. Male caregivers might benefit from cooking courses combined with nutritional guidance. |
| Puranen et al., (2015) | Finland | n = 40 Field notes and survey | Describing the process and feasibility of a randomised controlled trial of an intervention that provided nutritional guidance to home-dwelling people with AD. | 1 year | Assessment-based, tailored nutritional guidance implemented with a personal and positive approach may inspire and empower AD families to make positive changes in their diets. |
| Riviere et al., (1998) | France | n = 72 case-control study | To compare Vitamins C and E plasma levels in patients with AD and assess Vitamin C intake and nutritional status. |  | Mechanisms by which AD is associated with low plasma Vitamin C remains to be identified. |
| Riviere et al., (2001) | Europe | n = 224 Non randomised controlled trial | To determine if a nutritional education program prevents weight loss in AD patients. | 1 year | By giving nutritional information and support to families, it is possible to improve the patient’s global state of health. It is therefore important to assess the nutritional status and to follow the weight in patients, while giving support to caregivers. |
| Riviere et al., (2002) | Europe | n = 224 Cross-sectional observation study | Investigate the predictors of aversive feeding behaviours in people living with AD at home with a caregiver. | 1 year | It is important to develop interventions for caregivers and to assess, manage and treat the caregivers stress in order to prevent nutritional problems. Examining the interactional components of meals within the caregiving dyad is probably one of the best strategies to allow people with dementia to have adequate nutrients, especially in later stages of dementia. |
| Rullier et al., (2013) | France | n = 56 (caregiving dyads) cross-sectional study | The association of individual characteristics of people with dementia and family caregivers with nutritional status of people with dementia. | 6 months | Nutritional deficiencies in dementia should be investigated within the caregiving dyad and the nutritional status of CG should also be assessed. |
| Salva et al., (2009) | Spain | n = 946 Cluster randomised multi-centre study | Testing a socio-educative and nutritional intervention program to prevent weight loss and loss of function in dementia pts (NutriAlz) - published baseline characteristics | 1 year |  |
| Salva et al., (2011) | Spain | n = 946 Cluster randomised multi-centre study | Testing a socio-educative and nutritional intervention program to prevent weight loss and loss of function in dementia pts (NutriAlz). | 1 year | Patient specific oriented nutritional intervention and activity programmes. |
| Scarmeas et al., (2009) | America | n = 1393 Longitudinal follow-up study | Investigation into the adherence to the Mediterranean Diet and incidence of MCI and progression from MCI to AD. | average 4.3 years | Replication in other populations. Biological mechanisms involved still unclear. |
| Shatenstein et al., (2001) | Canada | n = 2427 Cross-sectional cohort study | Anthropometric differences in an elderly cohort - some who have dementia. |  | Simple anthropometric indicators would appear to be useful proxy measures for nutritional state, if more detailed info is unavailable.  Need for broad-based nutrition research in senior populations to elucidate predictors of poor nutritional status. |
| Shatenstein et al., (2007) | Canada | n = 72 Longitudinal follow-up study | To follow the natural evolution of dietary and nutrition status among elderly community dwelling adults with AD. | 1 year | This population would benefit from systematic dietary assessment and intervention to prevent further deterioration in food consumption and increased nutritional risk. |
| Shatenstein et al., (2008) | Canada | n = 2 Case Studies | The application of dietary intervention strategies in two participants of a larger study - one successful one unsuccessful. Provides understanding of what works/does not and why. | Full study was for 6 months | Patients should undergo full nutritional assessments as part of the review process at memory/geriatric clinics. Timely intervention is required. Nutrition professionals are needed in the setting where AD patients are treated. |
| Shatenstein et al., (2016) | Canada | n = 67 (caregiving dyads) Quasi-experimental controlled study | Measuring the effectiveness of a dietary intervention to improve nutritional status in community-dwelling AD patients. | 6 months | Diet must be assessed and tracked from diagnosis and the caregiver involved. |
| Silva et al., (2013) | Canada | n = 33 (caregiving dyads)  Semi-structured interviews (telephone) | To identify difficulties in dietary management encountered by caregivers and gather opinions on a nutrition intervention strategy. |  | A better understanding of the caregiver’s experience is essential for the development of nutrition interventions adapted to the needs of older adults with AD. |
| Smith et al., (1998) | America | n = 439 Longitudinal study | Patterns and associates of hyperphagia in community-based pts with dementia. | 18 months |  |
| Soto et al., (2012) | France | n = 414 Prospective cohort study | To explore whether weight loss is a predictor of rapid cognitive decline in people with AD. | 4 yrs | Weight assessment is simple to perform in current medical practice. |
| Suominen et al., (2015) | Finland | n = 99 recruited Randomised controlled trial | The effect of a tailored nutritional guide on nutrition, health-related Quality of Life, and falls in persons with AD. | 1 yr | Future studies ought to focus not only to weight loss but also to protein intake and other nutrients in order to maintain good nutritional status. Assessment-based, tailored nutritional guidance should be a significant part of the care of old and frail individuals. |
| Tombini et al., (2016) | Italy | n = 90 (caregiving dyads) Cross sectional study | Measuring nutritional status using MNA, comparing to caregiver nutritional status, and exploring the influence of different factors on nutrition. |  | Important to identify early signs of malnutrition in AD pts and caregiver's with a tool such as MNA. Nutrition Education should also be provided. |
| Tully et al., (2003) | Ireland | n = 193  Case control study | Determining n-3 PUFA status in free-living pts with AD using an established biomarker. |  | Further research into whether low DHA status in AD causes progression of disease. |
| Vellas et al., (2005) | France | n = 523 Prospective cohort study | To determine the impact of nutritional status on evolution of AD and on response to AchEI in people with AD living at home. | 1 year |  |
| Venci et al., (2015) | America | n = 60 Descriptive, cross-sectional study | To determine water intake and patterns of beverage consumption and contribution to total daily micronutrients and energy in older adults with memory decline. |  | Incorporating adequate amounts of beverages in meals and snacks may help older adults meet their nutrient recommendations. |
| Winograd et al., (1991) | America | n = 64 Cross sectional study | Nutritional intake in patients with senile dementia of the Alzheimer type. |  |  |
| Wlodarek et al., (2013) | Poland | n = 160 Cross sectional study | Assessment of the quality of diet of AD individuals living at home, Nursing Home vs. control. |  | Suitable nutritional intervention may have general positive impact on the diet of the elderly. |
| Wolf-Klein et al., (1995) | New York | n = 21 Cross sectional study | Measuring the resting energy expenditure in people with AD in different settings. |  | Further studies are needed to elucidate the mechanisms leading to potential altered body composition in pts with AD. For clinical practice authors recommend additional 200kcal/kg/day in addition to estimated energy requirements preferably as a snack. |

AD, Alzehimer’s Disease; MNA, Mini-Nutritional Assessment; PUFA, Polyunsaturated fatty acids; DHA, Docosahexaenoic acid; AchEI, acetylcholinesterase inhibitor; FTD, Frontotemporal dementia;

**Supporting Material Table 1.** Included studies following screening
